# Advances in nonparametric item response theory for scale construction in quality-of-life research

**DOI:** 10.1007/s11136-021-03022-w

**Published:** 2021-11-09

**Authors:** Klaas Sijtsma, L. Andries van der Ark

**Affiliations:** 1 Tilburg School of Social and Behavioural Sciences, Tilburg University, P.O Box 90153, 5000 LE Tilburg, The Netherlands; 2 Research Institute of Child Development and Education, University of Amsterdam, P. O. Box 15776, 1001 NG Amsterdam, The Netherlands

**Keywords:** Goodness of fit, Measurement of health-related attributes, Nonparametric item response theory, Rasch model

## Abstract

We introduce the special section on nonparametric item response theory (IRT) in *Quality of Life Research*. Starting from the well-known Rasch model, we provide a brief overview of nonparametric IRT models and discuss the assumptions, the properties, and the investigation of goodness of fit. We provide references to more detailed texts to help readers getting acquainted with nonparametric IRT models. In addition, we show how the rather diverse papers in the special section fit into the nonparametric IRT framework. Finally, we illustrate the application of nonparametric IRT models using data from a questionnaire measuring activity limitations in walking. The real-data example shows the quality of the scale and its constituent items with respect to dimensionality, local independence, monotonicity, and invariant item ordering.

## Introduction

This special section of *Quality of Life Research* is devoted to nonparametric item response theory (IRT) models [1]. In this introduction, we review nonparametric IRT models, provide references to more detailed texts, and show how the diverse set of papers in the special section fits into the nonparametric IRT framework. Nonparametric IRT models are generalizations of a large class of parametric IRT models including the Rasch model, the 2-parameter and 3-parameter logistic IRT models for binary item scores, and the partial credit model and the graded response model for polytomous item scores. Van der Linden [2] introduces these parametric IRT models extensively. Sijtsma and Van der Ark [3, Chap. 4] discussed how nonparametric and parametric IRT models are related in one large family of which the most general members are nonparametric IRT models. Parametric IRT models are special cases of nonparametric IRT models. Their generality renders nonparametric IRT models more flexible than most parametric IRT models.

IRT models are used for establishing whether a set of items intended to measure a particular attribute together constitute a scale for measurement. Examples of attributes are pain experienced by patients suffering from burn wounds [4], health-related quality-of-life aspects, such as physical functioning, general health perceptions, vitality, and social functioning [5], and adherence to medication and lifestyle for patients with hypertension [6]. Roorda et al. [7] used IRT models for scaling a set of items measuring activity limitations in rising and sitting down in patients with lower-extremity disorders living at home, and Sijtsma et al. [8] used nonparametric IRT models to analyze the World Health Organization Quality-of-Life scale (WHOQOL-Bref). In the special section, Feng et al. [9] applied nonparametric IRT models to the EQ-5D, a widely used generic measure of health, and based on the scaling results reinterpreted the EQ-5D scales. The number of articles using IRT models and other scaling techniques for constructing scales and assessing measurement quality *Quality of Life Research* published over the years is very large. It shows the paramount importance of well-founded measurement.

### Features or nonparametric and parametric IRT modeling

**Fig. 1:**
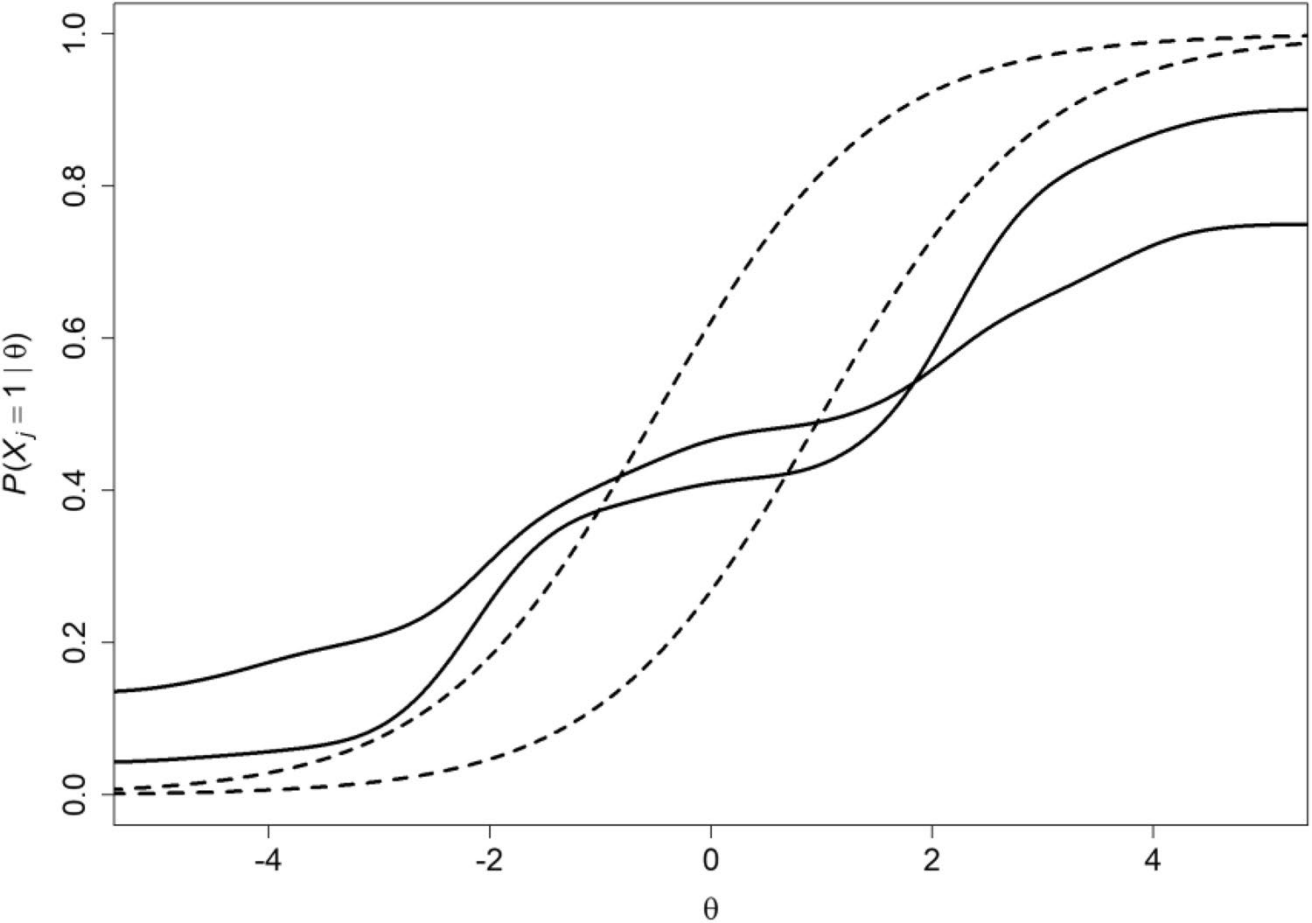
Four items having monotone IRFs consistent with the monotone homogeneity model, of which two (dashed logistic curves) also follow the Rasch model

**Fig. 2:**
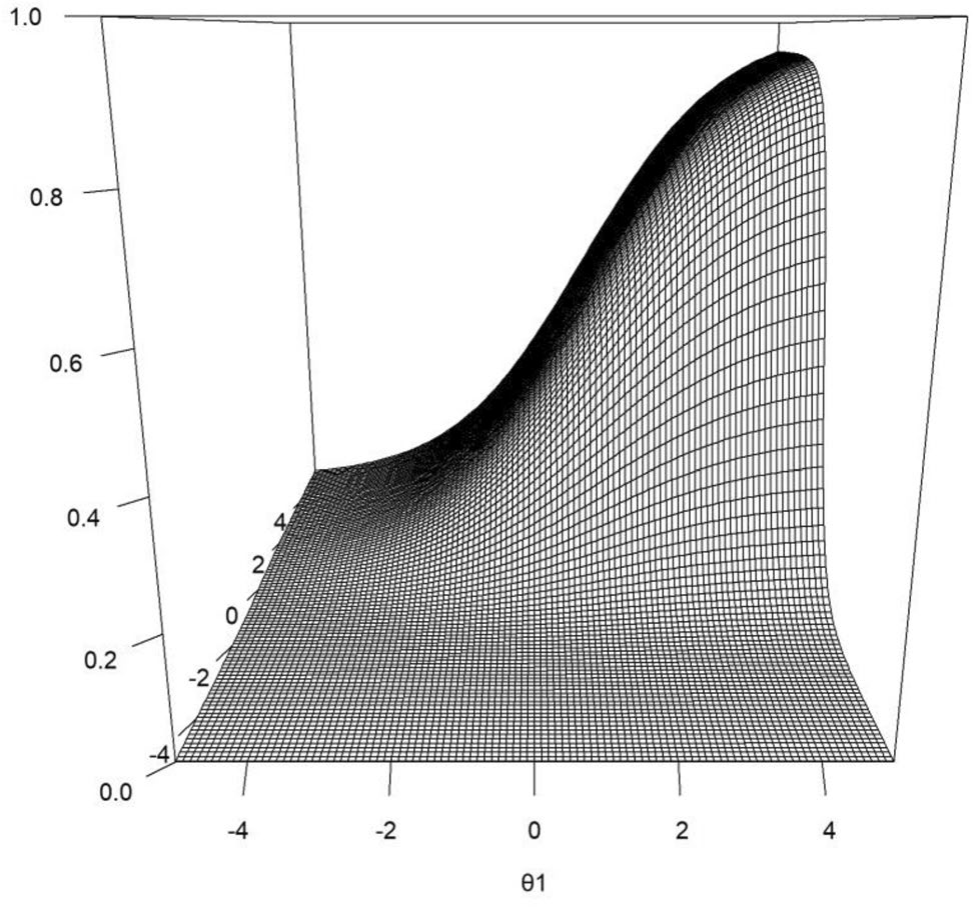
The IRF of a dichotomous item in a two-dimensional latent-variable model. The first latent variable (*θ*1) is on the *x*-axis, the second latent variable (*θ*2; label not shown) is on the *y*-axis, and the probability of obtaining item score 1 given the values of *θ*1 and *θ*2 is on the *z*-axis

The main difference between nonparametric and parametric IRT models is that the nonparametric models rest on assumptions about people responding to items in a test or a questionnaire that are more liberal than the assumptions parametric models make. For example, nonparametric IRT models assume that the relation between the probability of a patient giving a positive response to an item indicating ease of climbing the stairs and the underlying attribute of physical functioning is monotone—the better physical functioning, the more ease climbing the stairs—and parametric IRT models assume the relation not only is monotone but also logistic. This extra condition renders the relation more restrictive and the fit of the IRT model to the data more problematic. For example, the nonparametric model of monotone homogeneity [10; 1, Chap. 3] assumes monotone item response functions (IRFs), which can have any shape and intersect mutually (Fig. 1, all curves), and the logistic IRFs of the Rasch model all have the typical S shape while running parallel (Fig. 1, dashed curves). The models are equal in that sets of items comprising a test or questionnaire measure one attribute such as physical functioning and represented mathematically by one latent variable (typically denoted by $\theta$; Fig. 1). In addition, the models assume that there are no other attributes or covariates active affecting the covariances between item scores, so that inter-item covariances vanish when conditioning on the latent variable. These assumptions are unidimensionality and local independence, respectively. Parametric IRT models generalize to multiple latent variables thus allowing various attributes simultaneously to affect response probabilities (Fig. 2). Nonparametric IRT focuses on search algorithms to identify separate item clusters measuring different attributes or aspects of the same attribute.

**Fig. 3:**
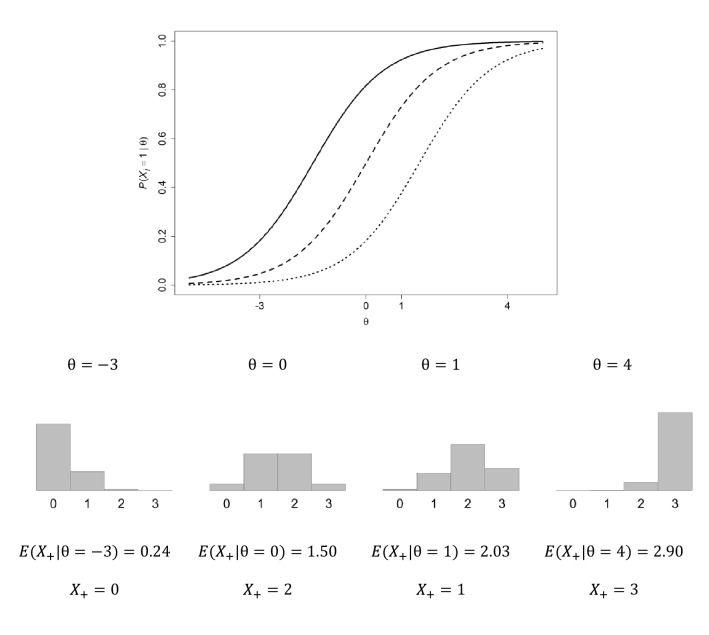
Upper panel: Three Rasch items (locations ${\delta }_{1}=-1.5$, ${\delta }_{2}=0$, ${\delta }_{3}=1.5$) and four example $\theta$-values (${\theta }_{1}=-3$; ${\theta }_{2}=0, {\theta }_{3}=1, {\theta }_{4}=4$) plotted on the horizontal axis. Three dichotomous items allow four sum scores: ${X}_{+}=0, 1, 2, 3$. Lower panel: Histograms showing the the sum-score distribution for each $\theta$ value, and the corresponding expected (i.e., mean) sum score, $E\left({X}_{+}|\theta \right)$. Expected sum scores $E\left({X}_{+}|\theta \right)$ have the same ordering as $\theta$-values. Last line: Sum-score values ${X}_{+}$ obtained by randomly drawing from the histograms. Unreliability causes different orderings of ${X}_{+}$ and $\theta$ in this particular draw

Other features render nonparametric IRT models interesting. First, for binary-item tests and questionnaires consistent with the model of monotone homogeneity, ordering persons by their number of positive scores (the total number of 1 scores or the sum score) is stochastically equal (i.e., with possible random violations) to ordering them by means of their latent-variable scores. This property says that one does not need the latent-variable scores for ordering persons and that simple sum scores will suffice albeit with random error (Fig. 3). This is a strong result, because it holds for scales when the model of monotone homogeneity fits the data well, and one need not estimate latent-variable scores at all. Because the Rasch model and the 2-parameter and 3-parameter logistic models are special cases of the model of monotone homogeneity, these parametric IRT models imply the ordering property using the sum score as well. Unlike nonparametric IRT models, parametric IRT models estimate the latent variable and assign $\theta$ estimates to persons. If the purpose is to order persons on a scale, it does not matter whether one uses the estimated $\theta$ or the sum score. Both scores are liable to unreliability due to random error, and may not perfectly reflect the error-free ordering of persons. Unlike nonparametric IRT models, parametric IRT models allow the assessment of scale-dependent measurement precision using the estimated $\theta$. For polytomous-item tests and questionnaires, the ordering of persons by their sum scores approximates their ordering by latent-variable scores quite well, but may contain small but unimportant distortions.

**Fig. 4:**
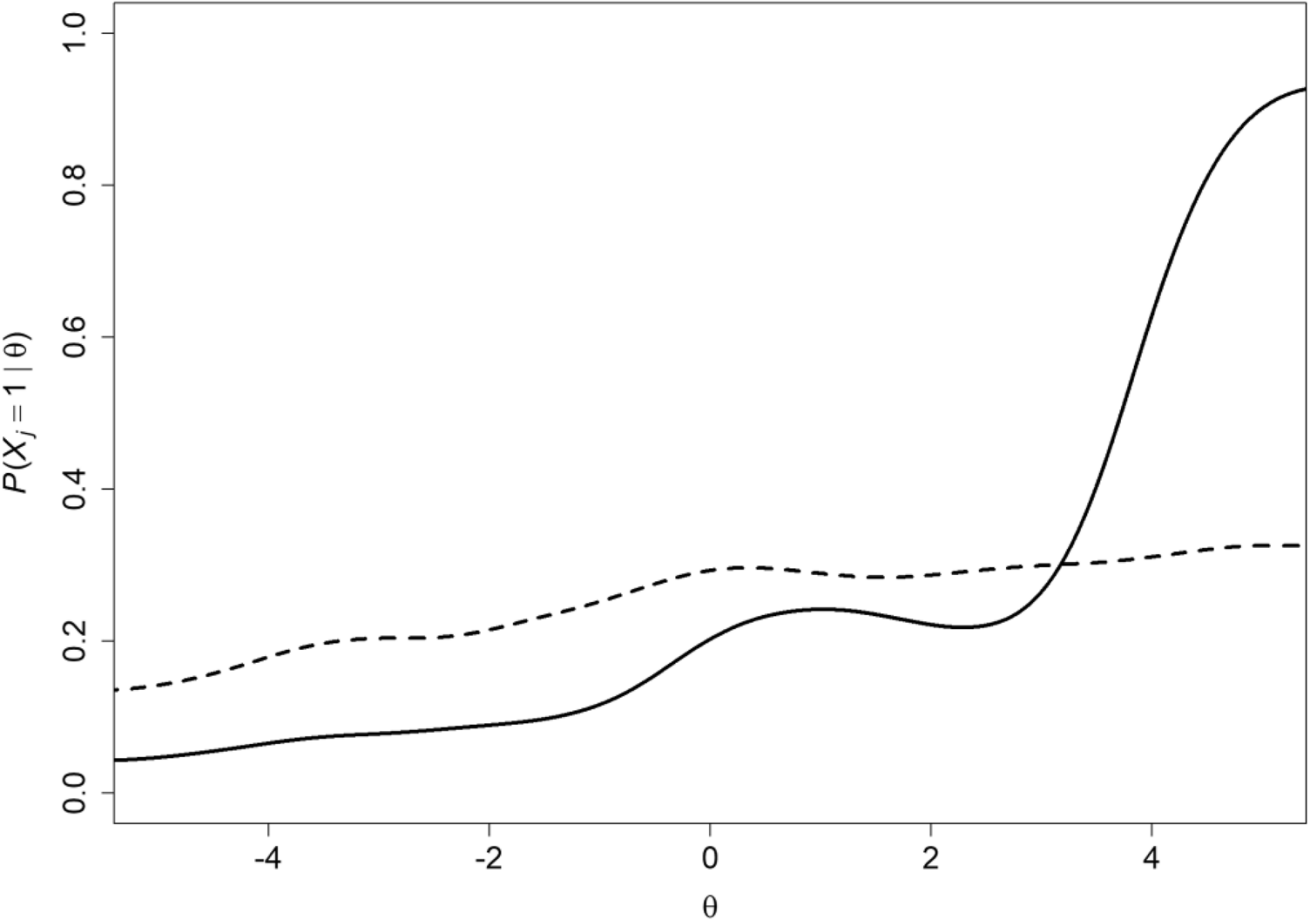
Two IRFs showing one item that works well only for people with high latent-variable levels (solid curve), and one IRF that has a weak and irregular relation with the latent variable and is a candidate for replacement (dashed curve)

Second, one might argue correctly that assuming logistic IRFs provides efficient item information by means of estimated difficulty, discrimination and perhaps pseudo-guessing parameters, but the fact that nonparametric IRT models do not commit to specific parametric IRFs, instead estimating the whole function for each item from the data allows a complete picture of item response behavior for each item. This allows the researcher to see that the item only works well for people high on the scale of, for example, physical functioning, but not for the majority (Fig. 4, solid curve), or that the IRF only has a weak and irregular relation with physical functioning and is a candidate for replacement (Fig. 4, dashed curve). Researchers and scale developers want to know these things and make decisions about maintaining, deleting or replacing items from preliminary tests or questionnaires. Only having estimates of where an item is located or whether it distinguishes people well at a particular scale location is useful but knowing the complete picture has greater diagnostic value for item assessment. Estimating IRFs of course is liable to sampling error and involves several rather arbitrary decisions [11].

Third, the fact that nonparametric IRT refrains from assumptions about response behavior that may be mathematically convenient but unnecessarily restrictive from a psychological or health-related point of view—which content-driven theory predicts that IRFs are S-shaped or run parallel—renders it more laborious when assessing whether the model is consistent with the data. Various methods exist for assessing IRF monotonicity and local independence and exploring the dimensionality of an item set, and several run into problems related to combinatorial explosions and the curse of dimensionality. They often provide a wealth of detail about goodness of fit, but have trouble summarizing these details into discrete often binary conclusions such as keeping an item or deleting it from the test or questionnaire. Parametric IRT models have the advantage of concentrating on a few parameters per item rather than estimating whole curves but at the basis of several of their goodness-of-fit statistics lie the observable data features that nonparametric IRT models explore in detail but find hard to summarize. Whereas nonparametric IRT sometimes struggles with all the details it wishes to involve, the level of granularity of parametric IRT may be too low, occasionally missing interesting data features. Of course, these characterizations are somewhat exaggerated to make our points but help to understand the topic of this article.

## Goodness of fit of models to data and robustness when models fail to fit

Sijtsma and Van der Ark [1] discussed a methodology in ten steps for investigating the goodness of fit of the nonparametric IRT models of monotone homogeneity and double monotonicity to the data collected with a (preliminary) test or questionnaire. The authors focus on the simple case of one test or questionnaire administered once to a sample from the population of interest. The first three steps concern aspects of data examination, in particular, recoding of item scores, handling of inadmissible and missing values and finally the identification and handling of outliers. These steps are useful in any scale analysis, not particular a nonparametric IRT analysis and we will skip them here. The next four steps numbered 4–7, concern scale identification and are highly relevant in the nonparametric IRT context. The authors claim that these four steps together identify one or more scales that satisfy the model of monotone homogeneity and, if desirable, investigate whether the identified scales also are consistent with the more restrictive model of double monotonicity that requires the monotone IRFs not to intersect. Both models enable a person ordering using the simple sum score and the latter model also enables an item ordering by item means that is the same, with the exception of possible ties, across the scale of the latent variable. The last three steps again are relevant to any scale analysis model, not only nonparametric IRT, and are reliability estimation for the sum score, determining norms tables for the interpretation of sum scores of individuals, and the comparison of scaling results across meaningful subgroups of the population in interest. Due to their generality, we will also skip these last three steps.

We illustrate the steps briefly using data from the physical health questionnaire Climbing Stairs (PHQ-CS) [12]. The 15-item questionnaire was administered to 759 subjects with lower-extremity disorders living at home. Each item consists of a statement that describes a problem a patient might encounter when climbing stairs, and the respondent either endorsed the statement (score 1) or not (score 0). The first 12 items pertain to 6 aspects of stair climbing (takes longer, different way, with difficulty, hold on to banister, use walking aid, helped by someone) that are applied to going up and going down, respectively. The last 3 items pertain to the frequency of climbing stairs. The complete PHQ-CS is available from [12].

### Step 4—scalability

A nonparametric IRT scale analysis usually starts with the determination of the dimensionality of the item set. That is, do we need one or more latent variables to explain the data structure and in case of multidimensionality, do the items divide neatly across two or more subclusters of items that are interpretable and form a basis for separate scales? Mokken [10] (also, see [13], Chap. 4) proposed an automated item selection algorithm based on scalability coefficient $H$ producing one or more preliminary scales in which both the individual items and the total item (sub)set satisfy minimum requirements for $H$ (called lower bounds). The requirements ascertain reliable person ordering on a scale spanned by the item (sub)set. Straat et al. [14] proposed an alternative item selection algorithm based on a genetic search. Brusco et al. [15], proposed an alternative clustering procedure. Zhang and Stout [16] discussed the DETECT procedure based on conditional covariances between item scores and Bolt [17] discussed related proposals. Van Abswoude et al. [18] used simulated data to compare various dimensionality assessment procedures. After determining a preliminary division of items in one or more item sets, for each set the assumptions of the nonparametric IRT models are investigated. In their contribution to the special section, Koopman et al. [19] discuss item selection based on scalability coefficients for clustered data common in much health research.

For the PHQ-CS, Mokken’s [10] automated item selection procedure produced the same results for lower bounds in the range from 0.0 to 0.4 (Table 1, columns 3 and 4): Except item 4, all items formed a single scale. Item 4 was excluded due to a negative item-pair scalability coefficient with item 12 (${\widehat{H}}_{4, 12}=-.058$; $\text{SE}=.417$). Because deleting item 4 did not alter the scalability, and because of the small point estimate and the large standard error, we decided to maintain item 4 in the scale. The other estimated item scalability coefficients were all larger than the conventional lower bound 0.3. The estimated scalability of the entire scale was $\widehat{H}=.497$ ($\text{SE}=.019$). Following Mokken’s [10] guidelines, $.4<H\le .5$ is a medium scale.

**Table 1:** Scaling results for PHQ-CS. Step 4 (Scalability) and Step 5 (Local Dependence): Automated item selection for lower bounds .0, .4, and .5; estimated item scalability coefficients (${\widehat{\text{H}}}_{\text{j}}$) plus standard error ($\text{SE}$) for the scale consisting of all 15 items; overview of positive locally dependent (PLD) item pairs

Item	Statement	Lower bound^a^			${\widehat{H}}_{j}$	SE	PLD item pairs^b^
		.0	.4	.5			
1	I go up the stairs but it takes longer	1	1	1	.618	(.024)	
2	I go up the stairs but in a different way	1	1	1	.447	(.025)	12
3	I go up the stairs but with (some) difficulty	1	1	2	.523	(.023)	
4	I go up the stairs and hold onto the banister			2	.576	(.039)	12
5	I go up the stairs and use a walking aid	1	1	1	.451	(.046)	11
6	I go up the stairs and am helped by someone	1	1	1	.418	(.097)	8, 10, 12
7	I go down the stairs but it takes longer	1	1	1	.578	(.023)	
8	I go down the stairs but in a different way	1	1	1	.437	(.026)	6, 12
9	I go down the stairs but with (some) difficulty	1	1	2	.527	(.023)	
10	I go down the stairs and hold onto the banister	1	1	1	.594	(.041)	6
11	I go down the stairs and use a walking aid	1	1	1	.506	(.044)	5
12	I go down the stairs and am helped by someone	1	1	1	.388	(.105)	2, 4, 6, 8
13	I do go up and down the stairs but less often	1	1	3	.419	(.026)	
14	I do go up and down the stairs but I avoid them	1	1	2	.447	(.027)	
15	I do go up and down the stairs but less stairs/floors	1	1	3	.425	(.032)	

Columns ‘Item’ and ‘Statement’ adapted from “Measuring activity limitations in climbing stairs: development of a hierarchical scale for patients with lower-extremity disorders living at home”, by Roorda et al. [12], Appendix. Copyright 2004 by Elsevier. Reprinted with permission

^a^1: item is selected into the first scale, 2: item is selected into the second scale; etc. Blank: item is unscalable

^b^If not a blank, the item may be in one or more positive locally dependent item pairs; the number indicates the other item in the positive locally dependent item pair(s)

### Step 5—local independence

To investigate the assumptions of nonparametric IRT models, one needs properties the models imply that do not contain the latent variable and can be computed directly from the data. An example is conditional association, and a special case of this property is the correlation between two item scores conditional on a function of the scores on one or more of the other items. Such a function is the sum score on these items, also called the rest score, which replaces the latent variable. These covariances must be nonnegative when the model of monotone homogeneity holds; negative values are inconsistent with the model. Straat et al. [20] used such conditional covariances to identify item pairs that are locally dependent, suggesting that their covariance does not only depend on the attribute one wishes to measure, but also on other, undesirable influences. Such items may be candidates for removal or replacement, or the researcher may maintain them when she assesses the inconsistency not serious enough. Also, see [21] for an approach based on nonparametric regression and the parametric bootstrap. For the PHQ-CS, using the method of conditional covariances [20] we detected several locally dependent item pairs (Table 1, last column). As this method was not investigated thoroughly, results should be interpreted with care [1].

### Step 6—monotonicity

To investigate whether response probabilities of a positive answer or picking a particular response category or a higher one are monotone related to the latent variable, again we need to assess a function the model of monotone homogeneity implies that does not contain the latent variable and can be computed directly from the data. Such a function conditions the response probabilities for an item on the sum score based on the other items, which is the rest score we discussed previously and replaces the latent variable. This is the observable property called manifest monotonicity [22]. Various nonparametric regression methods were proposed for assessing the monotonicity assumption, based on binning [23], kernel smoothing [11, 24], and spline fitting [25, 26]. Local decreases in estimated IRFs suggest that the item is ineffective for measurement at those scale ranges, but model-consistent local increases that have an almost flat slope may also be informative.

**Fig. 5:**
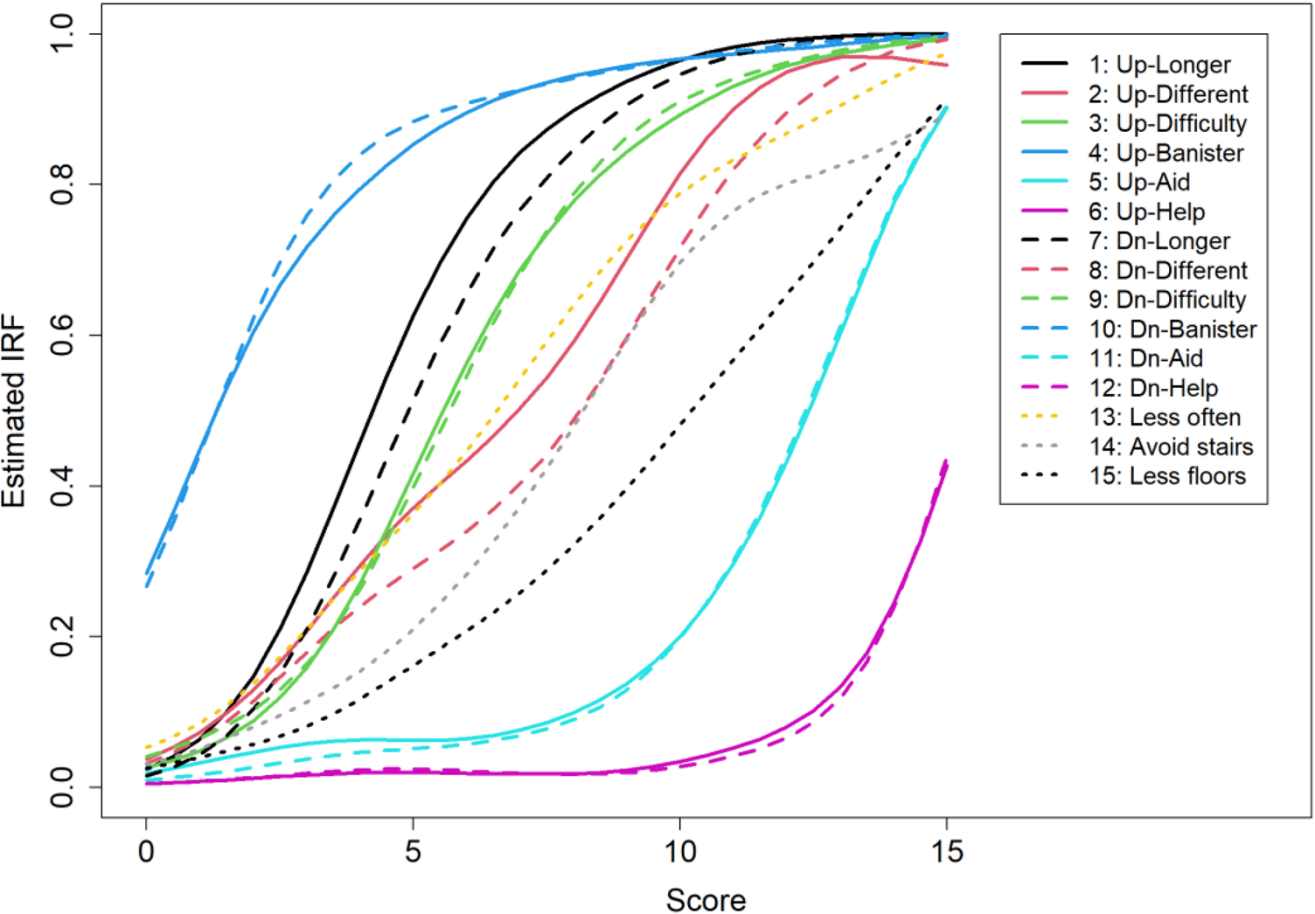
Estimated IRFs of 15 items from the PHQ-CS [12]. See Table 1 for the full item content

Figure 5 shows the estimated IRFs of the PHQ-CS. The IRFs were estimated using kernel smoothing setting bandwidth $h=.08$ [11]. Using the banister is the most popular coping strategy, whereas using assistance is the least popular. The IRFs of items pertaining to the same aspect of ascending and descending are remarkably similar. Items 2, 5, 11, and 14 show minor violations of monotonicity.

In their contribution to the special section, Falk and Fischer [27] study a flexible approach based on monotonic polynomials that provides a compromise by modeling items with both complex and simpler response curves. Their study investigates the suitability of items with IRFs described by monotonic polynomials for inclusion in patient-reported outcomes item banks.

### Step 7—invariant item ordering

**Fig. 6:**
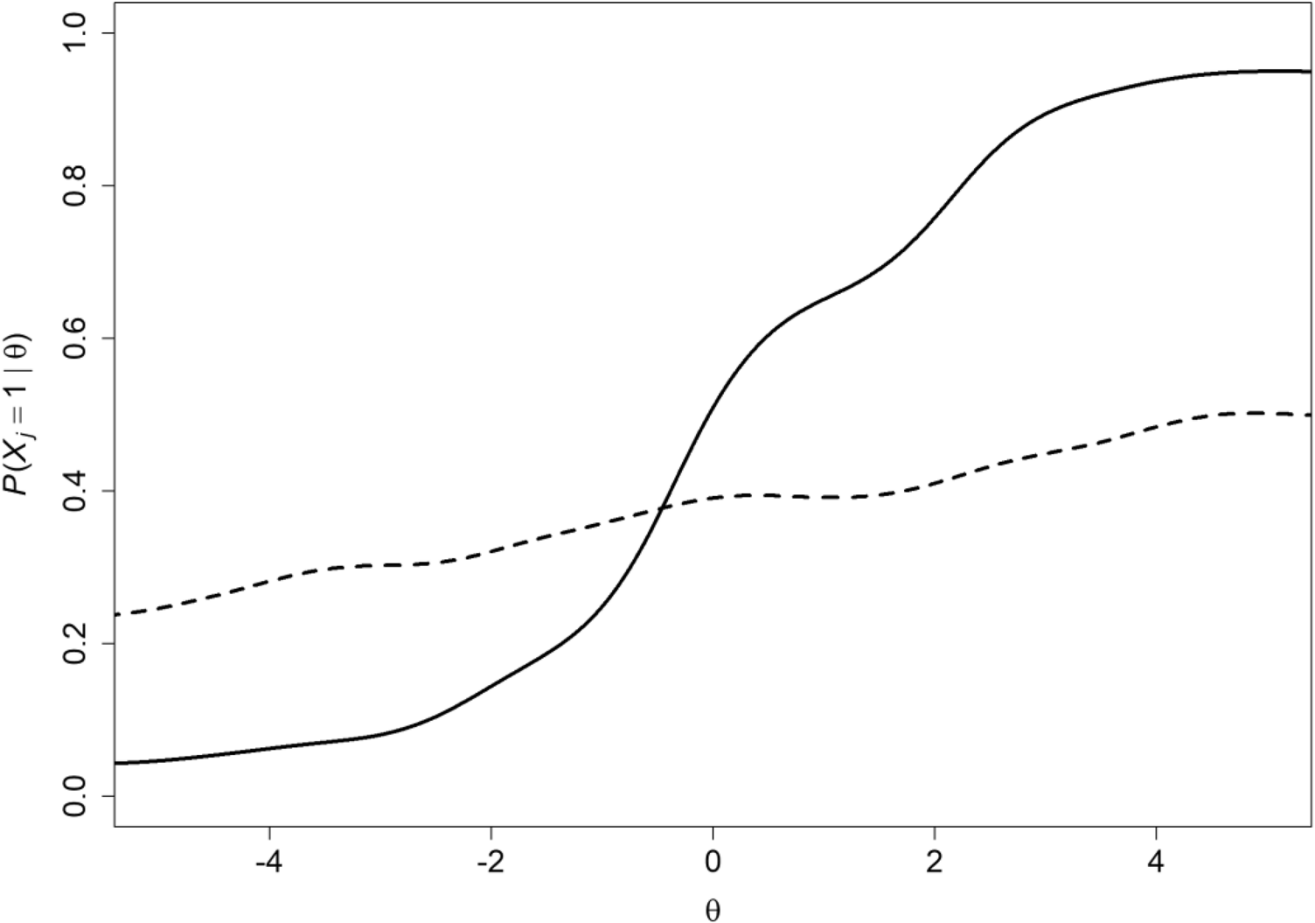
Two intersecting IRFs

The test is more informative when the ordering of the items by means of response probability or item mean score is the same, except for possible ties, for each measurement value. This means that if we know that for a particular scale value the probability of giving a positive response is greater for item $j$ than item $k$, we know that this ordering is the same—but never opposite—for all other scale values. This is different in parametric IRT models, where one often takes the ordering of items’ location parameters as their ordering according to difficulty or popularity, but when IRFs intersect, this is an incorrect conclusion (Fig. 6). Various methods exist for investigating whether sets of binary or polytomous items have an invariant ordering; see [28] for an automated methodology. Groundbreaking theoretical work was due to Rosenbaum [29], whereas Tijmstra et al. [30] provided new results.

Data analysis experience shows that an invariant item ordering is restrictive, rarely achieved for the whole set of items. For the PHQ-CS, an IIO is not achieved either, as the estimated IRFs in Fig. 5 cross. However, except for the item pair that relates to the aspect of ‘climbing in a different way’ (items 2 and 8), the pairs of items pertaining to the remaining five aspects seem to approximate an invariant ordering. ‘Holding on to the banister’ (items 4 and 10) is invariantly more popular than the remaining four aspects, ‘taking longer’ (items 1 and 7) is invariantly more popular than the remaining three aspects, ‘climbing with difficulty’ (items 3 and 9) is invariantly more popular than the remaining two aspects, and ‘using a walking aid’ (items 5 and 11) is invariantly more popular than ‘being helped by someone’ (items 6 and 12). The aspect ‘climbing in a different way’ is less popular than ‘using walking aids’ and more popular than ‘climbing with difficulty’ for the majority of patients, but not for the low-scoring patients.

When applied to real-data analysis, the previous four steps often provide detailed information on dimensionality, local independence, monotonicity, and invariant item ordering, and the researcher finds herself confronted with the question how to weigh and combine the information to draw a conclusion about the scale(s). In this special section, Crisan et al. [31] critically discussed a summary measure called *crit* that was proposed as a heuristic tool to help researchers finding their way in the output of a Mokken Scale Analysis [23]. The four analysis steps pertain to the situation in which a test or questionnaire is administered once to a sample from the population of interest, but in repeated measurement, the issue of response shift—that is, a change in patients’ perspective on the meaning of an item—may reveal itself through a change in item ordering at the individual level. In this special section, Dubuy et al. [32] mentioned chronic diseases where patients regularly adapt to their life circumstances, resulting in a different interpretation of items when tested repeatedly. In their contribution to the special section, these authors discuss a method to study this phenomenon of response shift for patient-reported outcomes.

## Discussion

Nonparametric IRT scaling puts fewer constraints on the data than several parametric IRT models do. This way, nonparametric IRT retains a larger number of items from preliminary test and questionnaire versions, which not only is efficient but also provides a good fit to the state of theory development for attributes in social, psychological, and health sciences. That is, theories for attributes may predict an attribute as cumulative (e.g., intelligence) or categorical (e.g., typologies), but theories do not (yet) predict that response probabilities for different items run parallel or can be described sufficiently well with one, two, or three parameters. In all fairness, data analysis experience has taught us that the data structure at best only approximates unidimensionality, local independence, monotonicity, and invariant item ordering, and there always is at least some discrepancy between model predictions and data structure. The crucial issue with all modeling attempts is not whether the model fits the data, but whether the discrepancy of model and data is small enough for the model properties to hold for the application at hand. Much research addresses whether statistical tests provide a Type I error that is almost equal to the significance level the researcher choses, and how particular data features not anticipated by the model affect a statistical test’s power. The question of the magnitude of discrepancy between IRT model and data is difficult to answer, because there are so many ways in which data can digress for the model’s prediction. Moreover, the practical use of a test or a questionnaire determines the cost or utility of false positives and false negatives in relation to correct decisions based on the sum score. The five articles in this special section provide valuable psychometric contributions to the further development of measurement in the health sciences.

## Data Availability

The data are not publicly available.

## Code availability

Computer code is available from the R package mokken [33, 34].
